# Rheumatism

**Published:** 1883-03

**Authors:** 


### HALL'S JOURNAL OF HEALTH

AND

MISCELLANY.

MONTHLY.

E. H. GIBBS, A. M., M. D., EDITOR.

FOUNDED IN 1854.

### RHEUMATISM.

WITH the advance of medical science, there has been a marked change in the views of physicians as to the causes and treatment of various diseases. A quarter of a century ago, bleeding was the principal remedy for rheumatism ; it was believed that the disease was owing to an excess of impure blood, and that it was desirable to get rid of as much of this vitiated fluid as possible, without destroying the life of the patient ; the older medical books, therefore, advised that bleeding should be carried to the extent of fainting, and repeated as soon as the patient had rallied sufficiently to endure it, until all pain had subsided. In conjunction with this treatmen large and often repeated doses of opium were prescribed with just enough ipecac and tartar emetic to keep the patient constantly nauseated, and make him as uncomfortable as possible ; the idea being that if a person was ill, the right thing to do was to make him worse—to tear down before building up. With all its absurdity, this plan had its advantages. It usually saved the patient from those complications that are often the result of the inflammation ; but if he survived it was apt to leave him with health permanently debilitated or broken.

The more advanced opinion as to the cause of rheumatism attributes it to an unhealthy condition of the secretions, owing principally to errors in diet. Not that all persons whose blood is impure have rheumatism ; but that the disease develops more readily in such persons when exposed to those changes of climate which in others produce only colds. There may be as large a percentage of rheumatic patients now as formerly, but we see fewer severe cases ; this is due, no doubt, to the more general diffusion of knowledge relating to the care of the health and the methods of avoiding disease.

Rheumatism, like nearly all other diseases, is at its commencement little more than an inconvenience that is easily arrested by simple remedies if applied promptly.

We have seen a sharp attack of rheumatism cured in one hour by keeping the painful part of the body as near the fire as could be borne, the object being to cause the blood to flow freely. The same thing may be accomplished by brisk rubbing ; hence the success of what is known as "the movement cure."

There is no doubt but that the condition of the system which favors the development of rheumatism, may be induced by the too free use of sugar, milk and starchy foods, as white flour and potatoes.

Beneficial results usually follow the almost total abstinence from such foods. If the quantities are diminished from pounds to ounces, there is still enough left to make an attractive bill of fare, in the way of brown bread, lean meats, poultry, fish, and nearly the entire list of fruits and vegetables.

As to clothing, too much importance can not be attached to the wearing of flannels summer and winter. They are the best protection against those sudden changes of temperature that so often mean mischief, whether rheumatism, pneumonia or other diseases. The greatest care should be taken to have the feet always dry and warm.

But the most important of all things in the prevention and cure of this and many other diseases is frequent and active exercise in the open air. The oxygen of the air we breathe is the greatest of all purifiers. The more out-of-door exercise we take the more air we breathe ; and the more air the greater is the purification of the blood, and the better the health. We had not intended to refer to the medical treatment of rheumatism ; but the disease is so common and the treatment so often unsatisfactory, that we are disposed to mention a few remedies that have seemed successful under our observation.

In case of local, chronic rheumatism, from a quarter to half a grain of morphine, administered hypodermically, has effected a complete cure in the half-dozen cases where we have employed it. One patient had suffered constantly for more than a year, and was cured in an hour in this way, and although fifteen years have elapsed, he is in robust health and has had not a twinge of rheumatism since. Another case in the same family, quite as severe, was healed in the same manner eight years ago, and there has been no return of the rheumatism. But this plan of treatment is not without danger, and should never be undertaken except by a competent physician. We have found the iodide of potassium of the most general application in rheumatism. From three to ten grains dissolved in half a tumbler of water and taken at bed time, and in severe cases in larger doses and oftener, will relieve most cases of rheumatism. Another remedy most highly commended is bicarbonate of potash 16 scruples; nitrate of potash, 8 scruples, water, one pint.—Dose a tablespoonful in half a tumbler of water 3 times a day, one hour before meals.

